# Supplementary material for: The effectiveness of peer-support for people living with HIV: A systematic review and meta-analysis
Source: PLoS One. 2021 Jun 17;16(6):e0252623. doi: 10.1371/journal.pone.0252623 (PMC8211296; doi:10.1371/journal.pone.0252623)
Supplement: S2 Table — Description of risk of bias assessment of each included study. (DOCX) [file pone.0252623.s002.docx]

S2 Table.

Risk of bias assessment of the included studies

**Brashers 2017**

| **Bias** | **Authors’ judgement** | **Support for judgement** |
| --- | --- | --- |
| Random sequence generation (selection bias) | Unclear risk | Quote: "Participants were randomly assigned into the treatment (Group 1) or control (Group 2) condition upon enrollment in the study." p.13. |
| Allocation concealment (selection bias) | Low risk | Quote: "The group assignment (Group 1 or Group 2) was placed with the questionnaires and schedule of activities for the study in a sealed envelope. When a participant was recruited, the recruiting nurse selected the next sealed envelope that contained the subject’s group assignment" p.13 |
| Blinding of participants and personnel (performance bias) | Unclear risk | Peer support intervention vs standard of care: Participants and peers not blinded. |
| Blinding of outcome assessment (detection bias) | Unclear risk | No blinding: outcomes self-reported. |
| Incomplete outcome data (attrition bias) | High risk | Appr. 21% loss (16 and 26%). Loss not described (e.g. reasons). No ITT |
| Selective reporting (reporting bias) | Low risk | Results section provide all outcomes stated in the methods section. No registration stated. |
| Other bias | Low risk | No other biases were found. |

**Broadhead 2012**

| **Bias** | **Authors’ judgement** | **Support for judgement** |
| --- | --- | --- |
| Random sequence generation (selection bias) | Unclear risk | Quote: "respondents were then randomly assigned to either Project CHAMPS (the PDI) or Project REACH (the UCI)" p.132 |
| Allocation concealment (selection bias) | Unclear risk | No information. |
| Blinding of participants and personnel (performance bias) | Unclear risk | Peer-driven intervention vs usual care intervention: Participants and peers not blinded. |
| Blinding of outcome assessment (detection bias) | Unclear risk | No blinding: outcomes self-reported. |
| Incomplete outcome data (attrition bias) | High risk | Loss over time similar in the two groups 30%, 30%, 48%, 50%. Loss not described (e.g. reasons). No ITT |
| Selective reporting (reporting bias) | Low risk | Results section provide all outcomes stated in the methods section. No registration stated. |
| Other bias | Low risk | No other biases were found. |

**Cabral 2018**

| **Bias** | **Authors’ judgement** | **Support for judgement** |
| --- | --- | --- |
| Random sequence generation (selection bias) | Unclear risk | Quote: "We randomized patients to each of the study groups using a parallel groups design" (p.2629) |
| Allocation concealment (selection bias) | Unclear risk | No information. |
| Blinding of participants and personnel (performance bias) | Low risk | Peer navigation and education intervention vs standard clinical care. Participants and peers not blinded but biological outcomes. |
| Blinding of outcome assessment (detection bias) | Low risk | No description of blinding, but biological outcome |
| Incomplete outcome data (attrition bias) | Low risk | Appr. 30% loss, balanced between groups, no description of reasons. ITT used. |
| Selective reporting (reporting bias) | Low risk | Protocol published that shows all outcomes are reported as planned. |
| Other bias | Low risk | No other biases were found. |

**Chang 2010**

| **Bias** | **Authors’ judgement** | **Support for judgement** |
| --- | --- | --- |
| Random sequence generation (selection bias) | Unclear risk | Quote: "We used an unrestricted randomization process. The 15 mobile clinic sites were randomized 2:1 to receive the PHW intervention  (Arms A, n =10 clusters) or control (Arm B, n= 5 clusters). We assigned clusters using unmatched, unrestricted random allocation by a drawing of lots. Study investigators (LWC, JK) generated the allocation sequence and implemented the randomization. This study was open label and unblinded" p.2 |
| Allocation concealment (selection bias) | Unclear risk | See above. |
| Blinding of participants and personnel (performance bias) | Low risk | Community-based peer health workers vs standard of care. Participants and peers not blinded but biological outcome |
| Blinding of outcome assessment (detection bias) | Low risk | No description of blinding, but biological outcome |
| Incomplete outcome data (attrition bias) | Low risk | Appr. 30% loss, balanced between groups, no description of reasons (except a few deaths). ITT used. |
| Selective reporting (reporting bias) | Low risk | Protocol published that shows all outcomes are reported as planned. |
| Other bias | Low risk | No other biases were found. |

**Chang 2015**

| **Bias** | **Authors’ judgement** | **Support for judgement** |
| --- | --- | --- |
| Random sequence generation (selection bias) | Low risk | Quote: “A list of random numbers with block sizes of 2, 4, and 6 was computer-generated” p.1743 |
| Allocation concealment (selection bias) | Low risk | Quote: “This allocation sequence was detailed on cards in sequentially numbered, opaque, sealed, and stapled envelopes. After completing the baseline survey, the next envelope was opened to obtain arm assignment” p.1743 |
| Blinding of participants and personnel (performance bias) | Low risk | Peer support vs standard of care. Participants and peers not blinded. Quote: “Participants were necessarily unmasked” p.1743, but biological outcome |
| Blinding of outcome assessment (detection bias) | Low risk | Quote: “Outcome assessment was also unblinded” p.1743 but outcome from clinical records |
| Incomplete outcome data (attrition bias) | Low risk | 0% loss (clinical records), 10%-12% loss (other). ITT and per-protocol analyses. |
| Selective reporting (reporting bias) | Low risk | Protocol published that shows all outcomes are reported as planned. |
| Other bias | Low risk | No other biases were found, except 6% of control participants received peer visits (contamination) |

**Coker 2015**

| **Bias** | **Authors’ judgement** | **Support for judgement** |
| --- | --- | --- |
| Random sequence generation (selection bias) | Low risk | Quote: “600 HIV-infected ART-naïve patients were randomized (1:1:1 ratio) to the 3 intervention groups. A blocked randomization method was used to generate  randomization schedules and individual-specific  randomization codes” p.280 |
| Allocation concealment (selection bias) | Unclear risk | No information. |
| Blinding of participants and personnel (performance bias) | Low risk | Peer education and home visit vs standard care. Participants and peers not blinded but biological outcome |
| Blinding of outcome assessment (detection bias) | Low risk | No description of blinding, but biological outcome |
| Incomplete outcome data (attrition bias) | High risk | 30% loss (28-31.5%) balanced between groups, no description of reasons (more men dropped out) |
| Selective reporting (reporting bias) | Low risk | Results section provide all outcomes stated in the methods section. No registration stated. |
| Other bias | Low risk | No other biases were found. |

**Cunningham 2018**

| **Bias** | **Authors’ judgement** | **Support for judgement** |
| --- | --- | --- |
| Random sequence generation (selection bias) | Low risk | Quote: “using sequentially numbered envelopes, we randomized the participants in a 1:1 ratio to the peer navigation intervention or transitional case management control group using computer generated,  randomly permuted blocks of 4 and 6 to prevent anticipation of assignment to study condition” p.e2 |
| Allocation concealment (selection bias) | Low risk | See above. |
| Blinding of participants and personnel (performance bias) | Low risk | Peer navigation intervention vs standard of care. Participants and peers not blinded but biological outcomes. |
| Blinding of outcome assessment (detection bias) | Low risk | No description of blinding, but biological outcome |
| Incomplete outcome data (attrition bias) | Low risk | 30% loss, balanced between groups, no description of reasons. ITT used. |
| Selective reporting (reporting bias) | Low risk | Protocol published that shows all outcomes are reported as planned (but 3 listed as secondary) |
| Other bias | Low risk | No other biases were found. |

**Cuong 2016**

| **Bias** | **Authors’ judgement** | **Support for judgement** |
| --- | --- | --- |
| Random sequence generation (selection bias) | Low risk | Quote: “Patients were allocated to the intervention group according to a randomization of clusters (communes) where patients lived...The randomization of matched clusters was through a computer software by a statistician not directly involved in the project. This study followed an open label cluster randomized controlled trial design” p.3 |
| Allocation concealment (selection bias) | Unclear risk | See above. |
| Blinding of participants and personnel (performance bias) | Low risk | Peer support vs standard of care. Participants and peers not blinded but biological outcome |
| Blinding of outcome assessment (detection bias) | Low risk | No description of blinding, but biological outcome (medical records) |
| Incomplete outcome data (attrition bias) | Low risk | 22% loss, balanced and reasons similar between groups. ITT used. |
| Selective reporting (reporting bias) | Low risk | Protocol published that shows all outcomes are reported as planned |
| Other bias | Low risk | No other biases were found. |

**Enriquez 2015**

| **Bias** | **Authors’ judgement** | **Support for judgement** |
| --- | --- | --- |
| Random sequence generation (selection bias) | Unclear risk | Quote: “Participants (n=20) were randomized to the peer-led adherence intervention (n=10) or …” p.3 |
| Allocation concealment (selection bias) | Unclear risk | No information. |
| Blinding of participants and personnel (performance bias) | Low risk | Peer-led HIV intervention vs ‘healthy eating’ control. Participants and peers not blinded but biological outcome |
| Blinding of outcome assessment (detection bias) | Low risk | No description of blinding, but biological outcome |
| Incomplete outcome data (attrition bias) | Low risk | 2 people in control group dropped out, but used ITT |
| Selective reporting (reporting bias) | Low risk | Results section provide all outcomes stated in the methods section. No registration stated. |
| Other bias | Low risk | No other biases were found. |

**Enriquez 2019**

| **Bias** | **Authors’ judgement** | **Support for judgement** |
| --- | --- | --- |
| Random sequence generation (selection bias) | Unclear risk | Quote: “participants signed consents, completed  baseline questionnaires, and were randomized to receive the Peers Keep It Real intervention immediately or were waitlisted. Participants in the wait-listed control group were to receive the standard of care for 6 months after enrollment” p.4 |
| Allocation concealment (selection bias) | Unclear risk | No information |
| Blinding of participants and personnel (performance bias) | Low risk | Peer support vs standard of care. Participants and peers not blinded but biological outcome |
| Blinding of outcome assessment (detection bias) | Low risk | No description of blinding, but biological outcome |
| Incomplete outcome data (attrition bias) | High risk | 23% loss, all in control group, no reasons provided |
| Selective reporting (reporting bias) | Unclear risk | No registration or protocol mentioned. Numbers and data of participants at follow up unclear. |
| Other bias | Low risk | No other biases were found. |

**Fogarty 2001**

| **Bias** | **Authors’ judgement** | **Support for judgement** |
| --- | --- | --- |
| Random sequence generation (selection bias) | Unclear risk | Quote: “158 women were randomly assigned to the standard group and 164 to the enhanced group” p.106 |
| Allocation concealment (selection bias) | Unclear risk | No information |
| Blinding of participants and personnel (performance bias) | High risk | Peer-based intervention vs standard care. Participants and peers not blinded, self-reported outcome |
| Blinding of outcome assessment (detection bias) | High risk | No blinding, self-reported outcome |
| Incomplete outcome data (attrition bias) | High risk | 21-40% loss, balanced and reasons similar between groups. No ITT |
| Selective reporting (reporting bias) | Low risk | Results section provide all outcomes stated in the methods section. No registration stated. |
| Other bias | Low risk | No other biases were found. |

**Giardano 2016**

| **Bias** | **Authors’ judgement** | **Support for judgement** |
| --- | --- | --- |
| Random sequence generation (selection bias) | Low risk | Quote: “Participants were randomized in a 1:1 allocation. Randomization was accomplished with a random number algorithm and was not blocked or stratified. J.D. maintained the randomization sequence, but that sequence was not linked to the study database until after unblinding” (supplementary) |
| Allocation concealment (selection bias) | Low risk | Quote: “the research coordinator opened a sealed intervention assignment envelope and contacted the appropriate intervention team” (supplementary) |
| Blinding of participants and personnel (performance bias) | Low risk | Peer mentor intervention vs instruction on safer sex. Participants and peers not blinded but biological outcome |
| Blinding of outcome assessment (detection bias) | Low risk | Quote: “the research coordinators responsible for follow-up interviews, the medical record reviewers, the data analyst, and the investigators, remained blinded until after the last participant completed follow-up for all outcomes” (supplemental) |
| Incomplete outcome data (attrition bias) | Unclear risk | 60% loss at 6 mo follow-up, used modified ITT |
| Selective reporting (reporting bias) | Low risk | Protocol published that shows all outcomes are reported as planned |
| Other bias | Low risk | No other biases were found. |

**Graham 2020**

| **Bias** | **Authors’ judgement** | **Support for judgement** |
| --- | --- | --- |
| Random sequence generation (selection bias) | Low risk | Quote:” Independent monitoring staff from the KEMRI Clinical Trials Unit used Stata to generate random assignments (1:1) within two strata: one for ART-naïve (62 numbered envelopes prepared) and one for ART-experienced men (34 numbered envelopes prepared). After enrollment by study staff, participants drew an opaque, sealed envelope from one of two bins: one for ART-naive and the other for ART experienced men” p.3 |
| Allocation concealment (selection bias) | Low risk | See above |
| Blinding of participants and personnel (performance bias) | Low risk | Peer support vs standard adherence counselling. Participants and peers not blinded but biological outcome |
| Blinding of outcome assessment (detection bias) | Low risk | Quote: “Laboratory staff assessing virologic outcomes were blinded to study arms” p.3 |
| Incomplete outcome data (attrition bias) | Unclear risk | 21-22% loss, balanced and reasons similar between groups (primarily transfer to other clinic). No ITT |
| Selective reporting (reporting bias) | Low risk | Protocol published that shows all outcomes are reported as planned |
| Other bias | Low risk | No other biases were found. |

**Liu 2018**

| **Bias** | **Authors’ judgement** | **Support for judgement** |
| --- | --- | --- |
| Random sequence generation (selection bias) | Low risk | Quote. “men consented to participate in Phase II trial  were randomized to receive either peer counselling or SOC within 1 week of their HIV diagnosis delivery. Treatment assignments generated by Vanderbilt Data Coordinating Center were put in numbered envelopes to be opened by study participants upon randomization” p.2 |
| Allocation concealment (selection bias) | Low risk | See above |
| Blinding of participants and personnel (performance bias) | Unclear risk | Peer counselling vs standard of care. Participants and peers not blinded. |
| Blinding of outcome assessment (detection bias) | Unclear risk | No blinding, self-reported outcome |
| Incomplete outcome data (attrition bias) | Low risk | 10%-55% loss, used ITT |
| Selective reporting (reporting bias) | Unclear risk | Protocol published that shows main outcome reported as CD4 cell count |
| Other bias | Low risk | No other biases were found. |

**McKirnan 2010**

| **Bias** | **Authors’ judgement** | **Support for judgement** |
| --- | --- | --- |
| Random sequence generation (selection bias) | Unclear risk | Quote: “317 (77%) agreed to enroll and were randomized to the comparison group (n=151) or intervention group (n=166)” p.957 |
| Allocation concealment (selection bias) | Low risk | Quote: “research assistant scheduled the consent and baseline interview and called a central research office to receive a randomly assigned participant number. The assigned identification number coded the participant as intervention or comparison” p.953 |
| Blinding of participants and personnel (performance bias) | Unclear risk | Peer support vs standard care. Participants and peers not blinded. |
| Blinding of outcome assessment (detection bias) | Unclear risk | No blinding, self-reported outcome |
| Incomplete outcome data (attrition bias) | Low risk | 20% loss, used ITT. |
| Selective reporting (reporting bias) | Low risk | Results section provide all outcomes stated in the methods section. Protocol published but wrong registration number so unable to check |
| Other bias | Low risk | No other biases were found. |

**Pearson 2007**

| **Bias** | **Authors’ judgement** | **Support for judgement** |
| --- | --- | --- |
| Random sequence generation (selection bias) | Low risk | Quote: “Random assignment to condition was based on a computer-generated allocation sequence prepared by an external statistician” p.3 |
| Allocation concealment (selection bias) | Low risk | Quote: “Allocation concealment involved the use of sequentially numbered, opaque, sealed envelopes containing the group assignment, which the research manager opened at the moment of randomization after enrolling participants” p.3 |
| Blinding of participants and personnel (performance bias) | Low risk | Peer support vs standard care. Participants and peers not blinded. Quote: “Due to the nature of the intervention, participants and the study team could not be blinded to intervention” p.3 |
| Blinding of outcome assessment (detection bias) | Low risk | Unblinded, see above, but biological outcome |
| Incomplete outcome data (attrition bias) | Unclear risk | 13.3%-25.7% loss, higher in standard care group, no information on reasons. ITT but some participants excluded |
| Selective reporting (reporting bias) | Low risk | Protocol published that shows all outcomes are reported as planned |
| Other bias | Low risk | No other biases were found. |

**Purcell 2017**

| **Bias** | **Authors’ judgement** | **Support for judgement** |
| --- | --- | --- |
| Random sequence generation (selection bias) | Low risk | Quote: “One staff member at each site who was not an intervention facilitator used a computer program to assign participants to 1 of the 2 intervention conditions” p.S36 |
| Allocation concealment (selection bias) | Low risk | See above |
| Blinding of participants and personnel (performance bias) | Low risk | Peer mentoring vs video discussion. Participants and peers not blinded but biological outcome |
| Blinding of outcome assessment (detection bias) | Low risk | No description of blinding, but biological outcome |
| Incomplete outcome data (attrition bias) | Low risk | Appr. 15% loss, balanced between groups |
| Selective reporting (reporting bias) | Low risk | Protocol published that shows all outcomes are reported as planned |
| Other bias | Low risk | No other biases were found. |

**Ruiz 2010**

| **Bias** | **Authors’ judgement** | **Support for judgement** |
| --- | --- | --- |
| Random sequence generation (selection bias) | Unclear risk | Quote: “A randomized, concurrent, follow-up study was conducted to...” p.411 |
| Allocation concealment (selection bias) | Low risk | Quote: “The doctor randomly (stratified by center) assigned patients to the corresponding intervention group, using an opaque, sealed envelope that had been delivered previously” p.411 |
| Blinding of participants and personnel (performance bias) | Low risk | Peer support vs standard care. Participants and peers not blinded but biological outcome |
| Blinding of outcome assessment (detection bias) | Low risk | No description of blinding (except statistician: “responses were evaluated by independent, blinded, personnel”) but biological outcome |
| Incomplete outcome data (attrition bias) | Low risk | 17%-19% loss, balanced and reasons similar between groups. Used ITT |
| Selective reporting (reporting bias) | Low risk | Results section provide all outcomes stated in the methods section. No registration stated. |
| Other bias | Low risk | No other biases were found. |

**Selke 2010**

| **Bias** | **Authors’ judgement** | **Support for judgement** |
| --- | --- | --- |
| Random sequence generation (selection bias) | Unclear risk | Quote: “This pilot prospective community randomized clinical trial…” p.484 |
| Allocation concealment (selection bias) | Unclear risk | Quote: “For each stratum, community names were placed in sealed opaque envelopes with 2 envelopes being assigned to the control group for every envelope assigned to the intervention group. Envelopes were then opened…” p.484 |
| Blinding of participants and personnel (performance bias) | Low risk | Peer support vs standard care. Participants and peers not blinded but biological outcome |
| Blinding of outcome assessment (detection bias) | Low risk | No description of blinding, but biological outcome |
| Incomplete outcome data (attrition bias) | Low risk | 8%-10% loss, balanced and reasons similar between groups |
| Selective reporting (reporting bias) | Low risk | Protocol published that shows all outcomes are reported as planned |
| Other bias | Low risk | No other biases were found. |

**Wouters 2014**

| **Bias** | **Authors’ judgement** | **Support for judgement** |
| --- | --- | --- |
| Random sequence generation (selection bias) | Unclear risk | Quote: “The random allocation sequence was generated by the principal investigator (researcher), using Stata’s sample command for selecting random samples from a population of observations without replacement.. The randomised control trial employed a Zelen-type double randomized consent design” (supplement) |
| Allocation concealment (selection bias) | Unclear risk | Quote. “lists with patient numbers was provided to the coordinators in charge of the implementation of the experiment, whom provided each of the peer adherence supporters with the relevant information” (supplement) |
| Blinding of participants and personnel (performance bias) | Low risk | Peer adherence support intervention vs standard of care. Participants and peers not blinded but biological outcome |
| Blinding of outcome assessment (detection bias) | Low risk | Quote: “Enumerators conducting the follow-up surveys and researchers collecting information from patient files at completion of the study were blinded after assignment” (supplement) |
| Incomplete outcome data (attrition bias) | High risk | 45%-68% loss. No ITT |
| Selective reporting (reporting bias) | Unclear risk | Protocol published that shows CD4 cell count and viral load should be reported |
| Other bias | Unclear risk | Some contamination between groups (supplement) |
