# Supplementary material for: The effectiveness of peer-support for people living with HIV: A systematic review and meta-analysis
Source: PLoS One. 2021 Jun 17;16(6):e0252623. doi: 10.1371/journal.pone.0252623 (PMC8211296; doi:10.1371/journal.pone.0252623)
Supplement: S1 File — (DOCX) [file pone.0252623.s003.docx]

# **The Review protocol:**

# **Effects of peer-support interventions for people living with HIV/AIDS: a systematic review with meta-analysis**

| Background |
| --- |
| Many people live with HIV/AIDS (PLWHA). Living with HIV/AIDS is challenging. PLWHA can be difficult to reach with ordinary health care services and can be defined as a vulnerable group. One possible solution to assist PLWHA is peer-support. Peer-support may provide social and emotional support and contribute to improved health outcomes. A number of studies have been carried out on peer-support interventions, of various design. Yet, the effects of peer-support interventions are unclear. We will conduct a systematic review that examines the effects of peer-support interventions for PLWHA. |
| Objectives |
| The aim of the systematic review is to examine the effects of peer-support interventions for people living with HIV/AIDS.  The review question is:  What is the effect of peer-support interventions for people living with HIV/AIDS? |
| Criteria for inclusion and exclusion of studies: |
| Types of studies |
| Eligible study designs are randomized-controlled trials (RCT), cluster-RCT, non-RCTs. |
| Types of population |
| People living with HIV/AIDS (PLWHA). In the event that an unmanageable number of studies are identified, we will only include studies with participants aged 18 years and older. Studies that focus on prevention of HIV and studies about mother-child transmission will be excluded. In the event that study populations are mixed (i.e. there are participants who are both under and over age 18) we will include the study when at least 50% are above age 18. |
| Types of interventions or interest |
| Peer-support intervention/programs. Our understanding of peer-support program is “the giving of assistance and encouragement by an individual considered equal” (Dennis 2003). To be included, peer-support must be given to an HIV positive person by another HIV positive person a minimum of 60 minutes face-to-face. In the event that we identify a large number of studies where peer-support is provided electronically, we will consider listing these studies, but not analyze them. This is for resource reasons and because an underlying premise of peer-support is that it works best with in-person interaction. All comparison conditions are eligible. |
| Types of comparison |
| All comparisons conditions are eligible. |
| Types of outcomes |
| Primary outcomes: Retention in care, antiretroviral therapy (ART) initiation, ART adherence, viral load, CD4 counts, quality of life and mental health. Secondary outcomes: adherence to care, HIV risk behaviors and stigma. Other related outcomes may be considered for inclusion. |
| Setting/context |
| We will include all settings. |
| Other inclusion criteria |
| Only studies published in English or Scandinavian languages (Norwegian, Swedish, Danish) will be included. We will include studies published after 1981, because this is the first year there were any publications about HIV/AIDS. |
| Search strategy for identification of studies |
| To identifying relevant keywords for the search, we conducted a preliminary literature search. An abbreviated PICO format was chosen as a framework for the search. The following Electronic Databases have been searched:  MEDLINE  MEDLINE In-Process  EMBASE CINAHL  PsycINFO  Other supplementary methods to identify relevant studies are: Screening of reference lists of the included studies and relevant literature reviews, citation searching and contacting experts in the field. We will conduct a search for grey literature in Scopus, Google Scholar, the BASE (Bielefeld Academic Search Engine), the UK government website and CORE. We will search for ongoing studies on clinicaltrials.gov and WHO Trial Register. |
| Method of Review |
| The systematic review will be conducted following the Cochrane Handbook for Systematic Reviews of Interventions (2019). |
| Selection of studies |
| Search records will be imported to EndNote and duplicates will be deleted. Search records will then be imported to Rayyan or a similar screening tool. Two reviewers will independently screen titles and abstracts of all identified studies. Studies considered relevant will be screened by two reviewers in full text. Pre-designed screening questions will be used to assure consistency. At each stage, the reviewers will discuss potential disagreements, and if no consensus is reached, a third reviewer will be involved until consensus is reached. Studies excluded after full-text consideration will be listed. |
| Assessment of methodological quality |
| Given the aim of this systematic review to examine the effect of peer-support interventions, we will appraise the controlled studies using design specific checklists. Two researchers will conduct independent appraisal and then agree on a final RoB evaluation. RCTs and C-RCTs will be appraised with the Cochrane Risk of Bias tool. Non-RCTs will be appraised with the EPOC Risk of Bias tool for N-RCTs. (https://epoc.cochrane.org/sites/epoc.cochrane.org/files/public/uploads/Resources-for-authors2017/suggested_risk_of_bias_criteria_for_epoc_reviews.pdf) |
| Data extraction |
| The data extraction will be carried out by one reviewer, and a second reviewer will check the completeness and accuracy of the data extracted. A data extraction form developed for the study will be used, to ensure standardization in data extracted. We will extract data regarding: publication characteristics (type of publication, author, year), study characteristics (e.g. country, study design, sample size), characteristics of the study participants (e.g. age, gender, co-morbidities, years living with HIV), characteristics of the providers/peer supporters (e.g. age, gender, training in peer-support, years living with HIV), characteristics of the peer-support (e.g. duration, content, setting, theoretical basis),characteristics of the control condition (e.g. duration, content, setting, theoretical basis) and study results (outcome data). Other data will be extracted as relevant. |
| Data synthesis |
| We will organize the studies according to comparisons. For each comparison we will evaluate the characteristics of the population (and intervention, comparison and outcome), and when they are considered sufficiently similar, we will conduct meta-analyses. When the populations of studies with the same comparisons are considered too different to pool statistically, we will report the results narratively. We will extract dichotomous and continuous data for all eligible outcomes where available. We will extract crude data and, when such data are available, adjusted outcome data (adjusted comparison (effect) estimates and their standard errors or confidence intervals). When information related to outcome measurement (e.g. sample sizes, exact numbers) is missing in the publication, we will contact the corresponding author(s) via e-mail and request the data. Results for the primary outcomes will be presented for each comparison. For meta-analyses we will use a random effects model. For dichotomous outcomes we will present the relative risk (or odds ratio) and the corresponding 95% Confidence Interval (CI). For continuous outcomes we will analyze the data using (standardized) mean difference ((S)MD) with the corresponding 95% CI. We will assess heterogeneity with I-square and Chi-square and assess the certainty of the evidence for each primary outcome using GRADE (Grading of Recommendations Assessment, Development, and Evaluation). |
| Timeframe |
| Anticipated to start May 2019 (search completed), anticipated completion date December 2020 |
