# Supplementary material for: The effectiveness of peer-support for people living with HIV: A systematic review and meta-analysis
Source: PLoS One. 2021 Jun 17;16(6):e0252623. doi: 10.1371/journal.pone.0252623 (PMC8211296; doi:10.1371/journal.pone.0252623)
Supplement: S2 File — (DOCX) [file pone.0252623.s004.docx]

**S2 File. Search strategy in MEDLINE**

MEDLINE via OVID, Date of search 11.01.2019. Database: Ovid MEDLINE(R) and Epub Ahead of Print, In-Process & Other Non-Indexed Citations, Daily and Versions(R) <1946 to January 08, 2019>. Search Strategy:

1 ((hiv or aids) adj6 (patient* or people* or person* or client* or living or men or women or woman or female* or adult* or service* or support* or positiv* or care or caring or affect*)).ti,ab. (168898)

2 peer group/ or peer influence/ (18925)

3 peer*.ti,ab. (77383)

4 (lay adj3 (people* or patient* or client*)).ti,ab. (1609)

5 (patient* adj2 expert*).ti,ab. (1932)

6 2 or 3 or 4 or 5 (86772)

7 1 and 6 (2341)

8 exp HIV Infections/ (266968)

9 exp Anti-Retroviral Agents/ (77058)

10 8 or 9 (291951)

11 (peer* adj6 (group* or support* or couns* or service* or provide* or care* or mentor* or tutor* or educat* or led)).ti,ab. (17653)

12 2 or 11 (31412)

13 10 and 12 (1666)

14 7 or 13 (2997)

15 ((hiv* or aids) and peer*).ti. (396)

16 14 or 15 (3042)

17 limit 16 to yr="1981 -Current" (3042)

Database: Ovid MEDLINE(R) ALL <1946 to May 26, 2020>. Search Strategy 28.05.2020:

1 ((hiv or aids) adj6 (patient* or people* or person* or client* or living or men or women or woman or female* or adult* or service* or support* or positiv* or care or caring or affect*)).ti,ab. (181191)

2 peer group/ or peer influence/ (20775)

3 peer*.ti,ab. (90039)

4 (lay adj3 (people* or patient* or client*)).ti,ab. (1787)

5 (patient* adj2 expert*).ti,ab. (2276)

6 2 or 3 or 4 or 5 (100163)

7 1 and 6 (2733)

8 exp HIV Infections/ (280882)

9 exp Anti-Retroviral Agents/ (82327)

10 8 or 9 (306972)

11 (peer* adj6 (group* or support* or couns* or service* or provide* or care* or mentor* or tutor* or educat* or led)).ti,ab. (20367)

12 2 or 11 (35235)

13 10 and 12 (1864)

14 7 or 13 (3420)

15 ((hiv* or aids) and peer*).ti. (455)

16 14 or 15 (3469)

17 limit 16 to yr="1981 -Current" (3469)
