# Supplementary material for: The effectiveness of peer-support for people living with HIV: A systematic review and meta-analysis
Source: PLoS One. 2021 Jun 17;16(6):e0252623. doi: 10.1371/journal.pone.0252623 (PMC8211296; doi:10.1371/journal.pone.0252623)
Supplement: Attachment — Submitted filename: Response to reviewers.docx [file pone.0252623.s005.docx]

04-April-2021

Manuscript PONE-D-21-00126 “Is peer-support for people living with HIV effective? A systematic review and meta-analysis”

PLOS ONE

REVIEWERS' SUGGESTIONS AND AUTHORS' ATTEMPT AT ADDRESSING THESE:

We are grateful to the two reviewers and the editor for stating that they find our manuscript to be interesting and well written. We appreciate their thoughtful comments and suggestions of ways to improve the quality of our manuscript. Their valuable insights have been carefully considered and integrated into the manuscript. We note that it was difficult to accommodate all suggestions, because for a few of the comments it was difficult to interpret what changes we were asked to do. We will be happy to attempt to change our text at a later point, with clarifications from the editorial office and/or reviewer(s).

JOURNAL REQUIREMENTS:

1. Please review your reference list to ensure that it is complete and correct.

Reponse: Reviewed as suggested. We believe the reference list is complete and correct.

2. Please ensure that your manuscript meets PLOS ONE's style requirements, including those for file naming.

Reponse: Checked as suggested. We believe the style requirements are met, including file naming.

3. Please attach a Supplemental file of the results of the individual components of the quality assessment, not just the overall score, for each study included.

Response: Provided as requested.

4. Please explain the reasons, and number of studies excluded for each reason, in the flow diagram.

Response: Provided as requested.

5. Please provide any updates you might have since the original search was performed in May 2020, or please provide the rational for ending your search at that time.

Response: We have not updated the search. Reviewing 5,470 records – and subsequent screening, RoB assessment, analyses, and manuscript write-up – is an enormous undertaking, which was started in June 2020. Upon submission in January 2021 and now in March 2021 the search is still less than 1 year old, which is considered current (Cochrane handbook chapter 4 section 4.4.10). It is unlikely that any relevant studies that would change our conclusions have been published since our search. However, in accordance with recommendations in the Cochrane handbook chapter 4, we plan to update the review when indicated.

6. Please include captions for your Supporting Information files at the end of your manuscript, and update any in-text citations to match accordingly.

Response: Provided as requested.

7. Thank you for stating the following in the Financial Disclosure section: "The author(s) received no specific funding for this work." We note that one or more of the authors are employed by a commercial company: PRAXIS, Kristiansand, Norway. a) Please provide an amended Funding Statement declaring this commercial affiliation, as well as a statement regarding the Role of Funders in your study. If the funding organization did not play a role in the study design, data collection and analysis, decision to publish, or preparation of the manuscript and only provided financial support in the form of authors' salaries and/or research materials, please review your statements relating to the author contributions, and ensure you have specifically and accurately indicated the role(s) that these authors had in your study. You can update author roles in the Author Contributions section of the online submission form. b) Please also include the following statement within your amended Funding Statement. “The funder provided support in the form of salaries for authors [insert relevant initials], but did not have any additional role in the study design, data collection and analysis, decision to publish, or preparation of the manuscript. The specific roles of these authors are articulated in the ‘author contributions’ section.” If your commercial affiliation did play a role in your study, please state and explain this role within your updated Funding Statement.

Response: We have provided an amended Funding Statement. This systematic review work was initiated and most of the work completed before Ms. Page was employed with PRAXIS (she was employed with the University of Agder while undertaking most of this work). Her work on this study was not part of her work as an employee of PRAXIS. PRAXIS had no role in this study: the organization did not play a role in the study design, data collection and analysis, decision to publish, preparation of the manuscript or financial support in the form of authors' salaries or research materials.

8. a) Please also provide an updated Competing Interests Statement declaring this commercial affiliation along with any other relevant declarations relating to employment, consultancy, patents, products in development, or marketed products, etc.  b) Within your Competing Interests Statement, please confirm that this commercial affiliation does not alter your adherence to all PLOS ONE policies on sharing data and materials by including the following statement: "This does not alter our adherence to PLOS ONE policies on sharing data and materials.” If this adherence statement is not accurate and there are restrictions on sharing of data and/or materials, please state these. Please note that we cannot proceed with consideration of your article until this information has been declared.

Response: We have provided an updated Competing Interests Statement declaring Ms. Page’s affiliation with PRAXIS. In the Statement we confirm that this commercial affiliation does not alter our adherence to all PLOS ONE policies on sharing data and materials by including the following statement: "This does not alter our adherence to PLOS ONE policies on sharing data and materials."

9. Please include both an updated Funding Statement and Competing Interests Statement in your cover letter. We will change the online submission form on your behalf.

Response: We have included an updated Funding Statement and Competing Interests Statement in our cover letter.

ADDITIONAL EDITOR COMMENT:

1. In case of high heterogeneity/statistical significant heterogeneity (e.g. adherence 6 and 12 months) no meta-analysis should be performed or heterogeneity should be explored (e.g. performing subgroup analyses) and plausibly explained why performing a meta-analysis is not misleading nevertheless.

Response: We quantified the degree of heterogeneity as follows (Higgins 2011): 0% to 40% might not be important. 30% to 60% may represent moderate heterogeneity. 50% to 90% may represent substantial heterogeneity. 75% to 100% considerable heterogeneity. Four of the meta-analyses evidenced high heterogeneity, shown with I^2^ and Tau^2^ (p<0.05): 1) Retention in care 6 mo. 2) Adherence to ART 12 mo. 3) Viral suppression 3 mo. 4) Viral suppression 12 mo. These four meta-analyses included two to four studies, thus limiting the possibility to perform subgroup analyses. We decided not to include the meta-analyses for Viral suppression 3 mo and Viral suppression 12 mo because subgroup analyses could not be performed. We explored reasons for heterogeneity for the other two meta-analyses and provide possible explanations (Retention in care 6 mo, Adherence to ART 12 mo).

REVIEWERS’ RESPONSES TO QUESTIONS:

Comments to the Author

1. Is the manuscript technically sound, and do the data support the conclusions? The manuscript must describe a technically sound piece of scientific research with data that supports the conclusions. Experiments must have been conducted rigorously, with appropriate controls, replication, and sample sizes. The conclusions must be drawn appropriately based on the data presented.

Reviewer #1: Yes

Reviewer #2: Yes

2. Has the statistical analysis been performed appropriately and rigorously?

Reviewer #1: Yes

Reviewer #2: Yes

3. Have the authors made all data underlying the findings in their manuscript fully available? The PLOS Data policy requires authors to make all data underlying the findings described in their manuscript fully available without restriction, with rare exception (please refer to the Data Availability Statement in the manuscript PDF file). The data should be provided as part of the manuscript or its supporting information, or deposited to a public repository. For example, in addition to summary statistics, the data points behind means, medians and variance measures should be available. If there are restrictions on publicly sharing data—e.g. participant privacy or use of data from a third party—those must be specified.

Reviewer #1: Yes

Reviewer #2: Yes

4. Is the manuscript presented in an intelligible fashion and written in standard English? PLOS ONE does not copyedit accepted manuscripts, so the language in submitted articles must be clear, correct, and unambiguous. Any typographical or grammatical errors should be corrected at revision, so please note any specific errors here.

Reviewer #1: Yes

Reviewer #2: Yes

5. Review Comments to the Author

Response: We thank the two reviewers for these positive evaluations.

REVIEWER 1

Overall: Very well-done review and meta-analysis with many important discussion points. I have minor suggestions overall and recommend publication.

1. Introduction: Only tiny changes: add an ‘s’ to word ‘setting’ on line 68.

Response: Revised as suggested.

2. ‘casted’ on line 117 should be ‘cast’

Response: Revised as suggested.

3. Methods: Line 179 – notes that a list of excluded studies can be requested – while I don’t think providing the list is necessary, it would be helpful to summarize the main reasons for exclusion, and if possible include the numbers of excluded for each reason (this could be included in Figure 1 if easiest)

Response: Added as suggested.

4. Results: I’m not seeing information in the text or Table 1 about which specific aspects of the included studies’ interventions included peer support. For example, what tasks did the peers typically take on as part of their roles? I think this will be of interest to readers and should be summarized somewhere, even briefly.

Response: Added as suggested.

5. I see Risk behaviors listed as an outcome 7 times in Table 1, but this is not included in your list of most commonly reported outcomes on line 262. Please adjust.

Response: Revised as suggested.

6. Line 366 - you mention the stigma outcome and reference [54], but I think you mean Wouters et al 2014 [55]? Also you say there was a number of ROBs for that reference, but I don’t see that as very evident in the ROB figure – there is more unclear rather than high risk necessarily (which is similar to some other studies, like Broadhead). It looks like only Fogarty had a number of high ROBs. Can you maybe explain the issues with that study a bit further, perhaps in the discussion? I’m wondering if they used a peculiar method of peer support to result in increased stigma.

Response: Wouters reference revised as suggested. Information about the study’s risk of bias revised as suggested. Discussion of Wouters included in discussion as suggested.

7. Line 355 – mentions ‘adherence to care’ – was this a different outcome from retention in care?

Response: Revised. Retention in care and adherence to care were separate outcomes. No studies reported on adherence to care (that was not ART adherence). Information added.

8. Discussion: line 370 you note similar reviews as [4, 20, 26-28, 36, 56] but 36 does not appear to be a review

Response: Revised as suggested.

9. I know of a few other reviews that look at peer support interventions for people living with HIV beyond the ones you cite (Boucher et al 2020, https://pubmed.ncbi.nlm.nih.gov/31598801/; Embuldeniya et al 2013, https://www.sciencedirect.com/science/article/pii/S0738399113000530?via%3Dihub), but of particular relevance may be Kanters et al 2016 which also includes a meta-analysis: https://onlinelibrary.wiley.com/doi/full/10.7448/IAS.19.1.21141 - Also it looks like some of your references to other reviews mention doing meta-analyses as well? Please ensure your text acknowledges how your meta-analysis relates to these others.

Response: Thank you for drawing our attention to these reviews. Embuldeniya et al. 2013 is a qualitative evidence synthesis (includes qualitative studies) on peer-support for individuals with chronic disease. Of 25 included studies, only 4 concerned individuals living with HIV. Boucher et al. 2020 investigates the effect of peer-led self-management intervention on ART adherence among people living with HIV (includes 13 studies from 2000-2018), with an emphasis on self-management (“definition arising from skill-building activities that are known to improve outcomes in self-management programs” page 1019). It has no meta-analyses. Because the interventions are somewhat different, there is limited overlap between the Boucher review and our review (only four studies are included in both). However, we now refer to this review. Kanters et al. 2016 examines the efficacy of “any peer-based intervention aimed to improve ART adherence on any HIV population” (page 2) in studies from 2004-2015. Given the differences in our inclusion criteria (particularly concerning population and intervention) there is limited study overlap in that review and our review (only three studies are included in both). Kanters et al., 2016 conducted meta-analyses (however, we note that there is no information on heterogeneity or follow-up time) and concluded peer-support leads to modest improvements in adherence to ART. We cite and refer to each of these reviews. Of the other related reviews we mention, only Medley et al. 2009 conducted meta-analyses, but this review addresses peer education for HIV prevention in developing countries (with studies from 1996-2006) and due to our different PICOs includes no studies that also we included, and the meta-analyses are on HIV knowledge, equipment sharing, condom use, STI prevalence.

10. I’m confused by this sentence on line 471: “The analysis demonstrated a yearly per patient costs of about $8.74 and $189 to avert one virologic failure.” – why are there 2 cost amounts but only 1 measure listed? Is it a range of costs for the measure? Please clarify.

Response: Revised as suggested.

11. line 472 – ‘through’ should be ‘though’

Response: Revised as suggested.

12. line 403 you say “our results indicate that different variations of peer support in different settings for different key populations appear effective. Presumably, this is because there is one or more common effective factors of the peer providing non-judgmental support, role-modeling, and personalized advice on daily activities and functioning.” – Following from this, I think it would be helpful to reiterate in the discussion the definition of peer support you used – e.g. minimum of 60 minutes face-to-face interaction etc. – because definitions vary widely. Also maybe some details from the studies if available (if not, it’s a common limitation). I think it is a strength and demonstrates the importance of personalized interaction (e.g. tailoring support to a person’s needs), which could also be useful to emphasize as recommendations for future peer support initiatives.

Response: Revised as suggested.

13. Conclusion doesn’t mention the improvement in viral suppression – would be good to add there too

Response: Revised as suggested.

14. Other: figures 7 and 9 – the “favours control” vs. “favours peer” x-axis labels order is flipped compared to all the other figures – should this be consistent with others?

Response: As per standard, in forest plots desirable and undesirable outcomes are presented differently.

15. Figure 8 – any reason for using green squares rather than blue?

Response: RevMan version 5.4, the Cochrane meta-analysis software we used, presents point estimates in green or blue depending on whether the outcome is continuous or dichotomous.

REVIEWER 2:

The study by Berg et al., with the title " Is peer-support for people living with HIV effective? A systematic review and meta-analysis ", aims to investigate the effectiveness of peer-support intervention on HIV care continuum and the quality of life of PLWH. The study is sound and is suggested to be the first to investigate the effect of peer-support on PLWH. The main objective of the study is to synthesize evidence on the effectiveness of peer-support intervention in the HIV community. This study could be relevant to policies maker and help improve the management of healthcare and lives of people living with HIV. However, the study showed some limitations, partly due to the inconsistency of the RCTs used for meta-analysis. Please see my comments below on some of these weaknesses.

Response: Thank you for your support of our study. We re-evaluated the homogeneity of the RCTs and decided against conducting some of the meta-analyses, due to statistical heterogeneity and possibly misleading results.

1. Please include the viral suppression failure in the abstract and discussion, as this was part of the meta-analysis.

Response: We are unsure what we are asked to change. We state results for viral suppression in the abstract and discussion. Results for virologic failure are not included in the abstract. This is due to word limits of the abstract and we elected to present results for the main outcomes with high certainty. We will be happy to change text related to virologic failure should the editorial office find that it would improve the paper.

2. Title: The author should consider revising the title to: " The effectiveness of peer-support for people living with HIV: A systematic review and meta-analysis".

Response: Revised as suggested.

3. The research question should be included in the introduction with the objectives and outcomes.

Response: Revised as suggested.

4. Abstract: The abstract should follow the Preferred Reporting Items for Systematic Reviews and Meta-Analyses (PRISMA) and include the objectives, outcomes and inclusion/exclusion criteria of the study.

Response: Revised as suggested to the extent possible relative to allowable word limit of the abstract (note that PRISMA states “Provide a structured summary including, *as applicable*: background; objectives; …).

5. Please include the viral suppression failure.

Response: See response 1 (reviewer 2).

6. Introduction: Please include reference in the second sentence.

Response: Revised as suggested.

7. Please change "Further" to "Furthermore", page 2, line 66.

Response: Revised as suggested.

8. Please add change "setting" to "settings", page 2, line 68.

Response: Revised as suggested.

9. Please add care to the following: "access to care", page 2, line 73.

Response: Revised as suggested.

10. Please remove "e.g" and just go with the citation, page 3, line 84.

Response: Revised as suggested.

11. Please correct to "Dennis et al.", page 3, line 98.

Response: Revised as suggested.

12. Please cite the book, page 3, line 99.

Response: Revised as suggested.

13. Please remove "e.g" and just go with the citation, page 4, line 113.

Response: Revised as suggested.

14. The author should clearly highlight the difference between this study and previous studies (Simoni and Genberg). The research question could be also stated here before the objectives. Clearly define the objectives, aims and anticipated outcomes of interest in the context of peer-support and this study.

Response: Revised as suggested.

15. Methods: Please change "Material and methods" to "Methods" or "Methods and Analyses"

Response: The headings are part of the journal’s manuscript body formatting guidelines (cannot be changed. We will be happy to change the heading should the editorial office find that it would improve the paper).

16. Please cite only, remove "as describe above", page 5, line 140.

Response: Revised as suggested.

17. Please use a less stigmatizing expression such as "People living with HIV- PLWH" instead of "HIV positive person". Restructure the sentence to: "....to be given to PLWH by PLWH for a minimum....", page 5, line 141.

Response: Revised as suggested.

18. Remove "(or viral suppression/failure)", page 5, line 146.

Response: We elected to keep the information (instead of deleting the info) in order to be transparent about acceptable outcomes.

19. Is adherence to ART a primary or secondary outcome?, page 5, line 147.

Response: We write “the following primary outcomes: retention in care, ART initiation, ART adherence...”. We believe it is therefore clear that ART adherence is a primary outcome.

20. Please remove "OVID" from all the databases, page 6, lines 157-160.

Response: We elected not to remove information on database service provider, because we wish to report transparently and the latest guideline statement asks that service provider is stated (McGowan J, et al. PRESS Peer Review of Electronic Search Strategies: 2015 Guideline Statement*. Journal of Clinical Epidemiology*. 2016, 75: 40-46.). However, we will be happy to remove this information should the editorial office find that it would improve the paper.

21. Search strategy: Please provide an example of the key words or combination of words used for the search. Appendix 3 doesn't show a specific example of search strategy.

Response: We are unsure what the reviewer means. Appendix 3 shows the exact search strategy used in Medline. All MeSH and keywords used are shown there.

22. Study selection. Please use Rayyan QCRI.

Response: Revised as suggested.

23. Please add a section for Outcomes (primary and secondary outcomes).

Response: We are unsure what we are asked to change. All outcomes are listed. We explain the data extraction and how we analyzed the data. We will be happy to change text or add a section related outcomes should the editorial office find that it would improve the paper.

24. Data analysis. Please keep reference [35] and delete the rest of the sentence: "for more information.........org".

Response: Revised.

25. Results: Delete line 232, page 9.

Response: Revised in accordance with the journal’s manuscript body formatting guidelines.

26. Delete line 269, page 14.

Response: Revised in accordance with the journal’s manuscript body formatting guidelines.

27. Delete line 286- 292, page 14

Response: Revised in accordance with the journal’s manuscript body formatting guidelines.

28. Please clarify this sentence: page 20, lines 304-305.

Response: We are unsure what we are asked to clarify. The retention in care outcome in Cabral et al. (time to 4-month gap in HIV care) could not be included in the meta-analysis. As stated in the text this is because Cabral et al. reported the outcome differently. The result in Cabral et al. was in favour of the intervention. We will be happy to modify the sentence should the editorial office find that it would improve the paper.

29. RR=1.06, 95% CI (1.01, 1.10) do not seem to be statistically significant. There seems to be no difference between both arms of the study, page 20, line 315. What was the P-value?

Response: We are unsure what the reviewer means. As shown, the CI is 1.01-1.10. Recall that the width of the confidence interval and the size of the p value are related, the narrower the interval, the smaller the p value. There is no p-value provided.

30. Please define primary and secondary outcomes in the "methods" section.

Response: Revised as suggested. We use the expressions as stated in the Cochrane Handbook and in accordance with the definition in: Andrade C. The primary outcome measure and its importance in clinical trials. J Clin Psychiatry. 2015.76(10):e1320-3.

31. Please include the number of excluded studies in the PRISMA flow diagram (Figure 1).

Response: Revised as suggested.

32. Although the RR and CI did not show strong evidence of the intervention effect, we seem to see a statistical significance with Retention in care (12 months), ART adherence (3 months) and viral suppression at 6 months (Figure 2, Enriquez and Graham, no meta- analysis) and virological failure (24 months), see Tables 2, 3, 5, and 7. Please discuss these findings more in the "discussion section". What does viral failure means in the context of peer-support?

Response: We agree that the results concerning retention in care, ART adherence, viral suppression, and virologic failure are noteworthy. Each are discussed in the discussion section, most quite extensively, thus we have not further expanded our rather lengthy discussion section (but we will be happy to do so should the editorial office find that it would improve the paper). The results regarding virologic failure were heterogeneous, but mostly favored peer-support. This means that people who receive peer-support are more likely to avoid viral failure.

33. Discussion. Please include the CI and the study significance in the discussion, page 22, line 374-376. Also include the reference Table.

Response: We are unsure what the reviewer means. Effect estimates and confidence intervals are not repeated in discussion sections, nor are references to results tables, and were we to include the 11 results suggested we would be in violation of the journal’s manuscript body formatting guidelines. However, we will be happy to modify the text should the editorial office find that it would improve the paper.

34. Please discuss the viral failure as well.

Response: Revised as suggested.

35. Please highlight the overall statistics of outcomes with significance.

Response: We are unsure what the reviewer means. All effect estimates are shown, with significance in terms of p-values and/or confidence intervals (in figures and/or tables). When an included study did not provide the group results, statistics and/or significance, we quote the result using text (in table 2).

36. Please discuss the results based on the evidences. Most of these RCT didn't show difference in the outcome (no significance) after the trial, page 23, lines 382-385.

Response: We are unsure what we are asked to change. The discussion of the results is based on the body of evidence, per Cochrane handbook guidelines (body of evidence is assessed with the Grading of Recommendations Assessment, Development, and Evaluation system). It would be in violation of systematic review guidelines and GRADE to vote count. In fact, the Cochrane handbook is clear that vote counting “should be avoided whenever possible” (section 9.4.11 in Handbook 5.1)

37. Please include CI and P-value, page 23, line 396 to 398.

Response: We are unsure what the reviewer means. Effect estimates and confidence intervals are not repeated in discussion sections, and including the CI and p-values would be in violation of the journal’s manuscript body formatting guidelines. However, we will be happy to modify the text should the editorial office find that it would improve the paper.

Author initiated changes: We previously stated that Enriquez et al. 2019 reported adherence to ART, but because HIV viral load suppression was used as the proxy measure of medication adherence ART, we state only ‘viral suppression’ in table 1.
