# Supplementary material for: The effectiveness of peer-support for people living with HIV: A systematic review and meta-analysis
Source: PLoS One. 2021 Jun 17;16(6):e0252623. doi: 10.1371/journal.pone.0252623 (PMC8211296; doi:10.1371/journal.pone.0252623)
Supplement: Attachment — Submitted filename: Response to reviewers.docx [file pone.0252623.s006.docx]

14-May-2021

Manuscript PONE-D-21-00126R1 “The effectiveness of peer-support for people living with HIV: A systematic review and meta-analysis”

PLOS ONE

EDITOR'S SUGGESTIONS AND AUTHORS' ATTEMPT AT ADDRESSING THESE:

Please review your reference list to ensure that it is complete and correct.

Response: We reviewed as suggested. We believe the reference list is complete and correct.

The list of excluded studies should be provided as supplemental material. Alternatively, you might delete "and studies excluded after full-text consideration were listed."

Response: We revised the sentence as suggested (deleted the clause).

Thank you for addressing heterogeneity in the meta-analyses. However, the explanations and rational for excluding studies from the analyses should be guided by clinical aspects rather than statistical reasons (e.g. "This was due to the large positive effect"), i.e. you should give possible clinical reasons why the effects of some studies differ. Excluding studies only because they cause statistical heterogeneity is not adequate.

Response: We conducted this systematic review and meta-analyses of effect in accordance with the *Cochrane Handbook of Reviews of Interventions*. For transparency, the sub-chapters from chapter 10 that are relevant to heterogeneity are pasted below. The Cochrane handbook distinguishes between three types of heterogeneity: clinical diversity, methodological diversity, statistical heterogeneity. It explains that when heterogeneity is identified among a group of studies that would otherwise be considered suitable for a meta-analysis, authors must take into account the statistical heterogeneity when interpreting results, particularly when there is variation in the direction of effect. In addition to general recommendations, the handbook explains seven options to handle heterogeneity: (1) Check again that the data are correct. (2) Do not do a meta-analysis. (3) Explore heterogeneity. (4) Ignore heterogeneity. (5) Perform a random-effects meta-analysis. (6) Reconsider the effect measure. (7) Exclude studies. We checked that the data were correct (option 1), discarded the option to ignore heterogeneity (option 4), conducted random-effects analyses (option 5), checked that the effect measures were correct (option 6). In our previous submission, we had opted for excluding one study from each analysis (option 7). We opted to do so because, when performing analyses both with and without these outlying study results, we observed that excluding the study either removed or significantly reduced heterogeneity. According to the editorial comment, "excluding studies is not adequate". That left us with two options of dealing with the two meta-analyses with high statistical heterogeneity. With respect to option 2 (do not do a meta-analysis), we considered it, but decided this was not the best option because only one study showed variation in results for the outcomes Retention in care 6 mo and ART adherence 12 mo. Additionally, we believe the trials are trying to measure essentially the same thing and that it is worth summarizing the result. With respect to option 3 (explore heterogeneity), there were too few studies to conduct subgroup analyses or meta-regression, and we had not pre-specified any such analyses, and conclusions from such analyses are nonetheless considered questionable. We therefore attempted to determine the cause of heterogeneity among the studies’ results by inspecting the characteristics (SPICO) of the studies. We were unable to explain the statistical heterogeneity based on clinical diversity and methodological diversity. Had there been clinical diversity and/or methodological diversity, we would have seen high statistical heterogeneity in also the other meta-analyses that included these studies. Specifically: Retention in care 6 mo has high statistical heterogeneity when all three studies are included (Cunningham, Giordano, Purcell) and there is no statistical heterogeneity when Cunningham is removed. We found that Cunningham is an outlying study regarding this specific outcome. This is because heterogeneity is high *only* in this meta-analysis with Cunningham: Cunningham does not cause statistical heterogeneity in Retention in care 12 mo (despite weighing more in this analysis) and not in the outcome Adherence to ART (neither when including all studies, nor when limiting the meta-analysis to just the same studies as in Retention in care). Examining the outcome result of Cunningham in Retention in care 6 mo shows that Cunningham has a greater positive result than the other studies. Similarly, Adherence to ART 12 mo has high statistical heterogeneity when all four studies are included (Cunningham, Pearson, Purcell, Selke) and heterogeneity is greatly reduced when Selke is removed. We found that Selke has an outlying study result regarding this specific outcome. This is because statistical heterogeneity is high *only* in this meta-analysis with Selke: Selke neither causes statistical heterogeneity in Adherence to ART 6 mo (despite weighing more in this analysis) nor in the outcome Virologic failure 12 mo. These examinations, and examinations of the other studies in these two analyses, strongly suggest that statistical heterogeneity is not due to the studies’ clinical or methodological diversity; if it were, statistical heterogeneity would be high also in the other meta-analyses that included these studies. In sum, we believe the group of studies reporting Retention in care and ART adherence are suitable for a meta-analysis: they are trying to measure essentially the same thing and it is worth summarizing the result. There is, however, high heterogeneity. Our examinations show that one study in each analysis causes the statistical heterogeneity, but we are unable to explain why, beyond that it has an outlying study result. Given the editorial team is reluctant to allow removal of an outlying study (option 7), we appear to be left with the option of reporting a meta-analysis with high heterogeneity that we attempt to explain (option 3), but cannot fully explain. In our opinion, this is acceptable, because we follow the Cochrane Handbook suggestion to take statistical heterogeneity into account when interpreting the results. We do so in presenting the results, discussing the results, and in our GRADE assessment. Importantly, the GRADE assessment is the final and crucial assessment of the results of the systematic review and the GRADE assessment (certainty of evidence) for these two outcomes is the same, whether there is no meta-analysis, meta-analysis with all possible studies, or meta-analysis with all minus one study included (see example GRADE tables below: we downgrade the result due to inconsistency). The difference is the ability to estimate pooled absolute and relative effects. As described by Melson et al. (2014) "The appraisal of the similarity of studies with regard to clinical and methodological heterogeneity and the ultimate decision of whether to include (or exclude) a certain study in a meta-analysis are the responsibility of the meta-analysts. As there are no criteria with which to quantify clinical and methodological heterogeneity, this appraisal is subjective." Both the Cochrane Handbook and Melson et al. (2014) also explain there is no uniform approach to dealing with heterogeneity and that statistical heterogeneity in the results does not mean one must abandon the meta-analysis, rather that one should interpret the results with caution. We are interpreting the results with caution. Here, we submit a new manuscript with no studies removed, but we are happy to submit a revised manuscript with one study removed, or a third option if we have missed any reasonable options of dealing with statistical heterogeneity in the two results. As an aside, but also important background information, is that the first author of this systematic review has authored several dozen systematic reviews, the first author’s full-time job is to supervise teams of reviewers who conduct systematic reviews and health technology assessments (HTA), and we consulted with a statistician whose job is to conduct and advise on meta-analyses at an HTA center.

Melson et al. The effects of clinical and statistical heterogeneity on the predictive values of results from meta-analyses. Clinical Microbiology and Infection. 2014. 20(2):123-129.

| **Table: Retention in care (3 studies in metaanalysis)** | | | | | | |  |
| --- | --- | --- | --- | --- | --- | --- | --- |
| **Outcome,**  **follow-up time** | **Anticipated absolute effects* (95% CI)** | | **Relative**  **effect**  **(95% CI)** | **No. of**  **participants  (Studies)** | **Quality of evidence (GRADE)** |  | |
|  | Assumed risk with control | Assumed risk with peer-support |  |  |  |  |  |
| Retention in care (6 mo) | 64.6% | 67.1% | RR=1.05 (0.92, 1.20) | 1916  (4 RCTs) | ⨁⨁⨁◯  MODERATE ^1,2^ |  | |
| 1. Downgraded by 1 level because of inconsistency.  2. 1 other RCT found a stat.sign. difference in favour of the intervention (see Tab 2) | | | | | | | |

| **Table: Retention in care (2 studies in metaanalysis)** | | | | | | |  |
| --- | --- | --- | --- | --- | --- | --- | --- |
| **Outcome,**  **follow-up time** | **Anticipated absolute effects* (95% CI)** | | **Relative**  **effect**  **(95% CI)** | **No. of**  **participants  (Studies)** | **Quality of evidence (GRADE)** |  | |
|  | Assumed risk with control | Assumed risk with peer-support |  |  |  |  |  |
| Retention in care (6 mo) | RR=0.98 (0.91, 1.07) to RR=1.16 (1.05, 1.28). See Fig 3 and Tab 2. | | | 1916  (4 RCTs) | ⨁⨁⨁◯  MODERATE ^1,2^ |  | |
| 1. Downgraded by 1 level because of inconsistency.  2. 1 other RCT found a stat.sign. difference in favour of the intervention (see Tab 2) | | | | | | | |

| **Table: ART adherence (4 studies in metaanalysis)** | | | | | | |  |
| --- | --- | --- | --- | --- | --- | --- | --- |
| **Outcome,**  **follow-up time** | **Anticipated absolute effects* (95% CI)** | | **Relative**  **effect**  **(95% CI)** | **No. of**  **participants  (Studies)** | **Quality of evidence (GRADE)** |  | |
|  | Assumed risk with control | Assumed risk with peer-support |  |  |  |  |  |
| ART adherence (12 mo) | 87.3% | 92.1% | RR=1.05 (0.98, 1.12) | 1140  (4 RCTs) | ⨁⨁⨁◯  MODERATE ^3^ |  | |
| 3. Downgraded by 1 level because of inconsistency. | | | | | | | |

| **Table: ART adherence (3 studies in metaanalysis)** | | | | | | |  |
| --- | --- | --- | --- | --- | --- | --- | --- |
| **Outcome,**  **follow-up time** | **Anticipated absolute effects* (95% CI)** | | **Relative**  **effect**  **(95% CI)** | **No. of**  **participants  (Studies)** | **Quality of evidence (GRADE)** |  | |
|  | Assumed risk with control | Assumed risk with peer-support |  |  |  |  |  |
| **ART adherence** | | | | | | |  |
| ART adherence (12 mo) | RR=0.97 (0.91, 1.03) to RR=1.07 (1.02, 1.13). See Fig 5 and Tab 2. | | | 1140  (4 RCTs) | ⨁⨁⨁◯  MODERATE ^3^ |  | |
| 3. Downgraded by 1 level because of inconsistency. | | | | | | | |

**Cochrane handbook**

**10.10.1 What is heterogeneity? #section-10-10-1**

Inevitably, studies brought together in a systematic review will differ. Any kind of variability among studies in a systematic review may be termed heterogeneity. It can be helpful to distinguish between different types of heterogeneity. Variability in the participants, interventions and outcomes studied may be described as **clinical diversity** (sometimes called clinical heterogeneity), and variability in study design, outcome measurement tools and risk of bias may be described as **methodological diversity** (sometimes called methodological heterogeneity). Variability in the intervention effects being evaluated in the different studies is known as **statistical heterogeneity**, and is a consequence of clinical or methodological diversity, or both, among the studies. Statistical heterogeneity manifests itself in the observed intervention effects being more different from each other than one would expect due to random error (chance) alone.

**10.10.3 Strategies for addressing heterogeneity#section-10-10-3**

Review authors must take into account any statistical heterogeneity when interpreting results, particularly when there is variation in the direction of effect (see [MECIR Box 10.10.b](https://training.cochrane.org/handbook/current/chapter-10#_Ref522111064)). A number of options are available if heterogeneity is identified among a group of studies that would otherwise be considered suitable for a meta-analysis.

1. *Check again that the data are correct.* Severe apparent heterogeneity can indicate that data have been incorrectly extracted or entered into meta-analysis software. For example, if standard errors have mistakenly been entered as SDs for continuous outcomes, this could manifest itself in overly narrow confidence intervals with poor overlap and hence substantial heterogeneity. Unit-of-analysis errors may also be causes of heterogeneity (see [Chapter 6, Section 6.2](https://training.cochrane.org/handbook/current/chapter-06#section-6-2)).
2. *Do not do a meta-analysis.* A systematic review need not contain any meta-analyses. If there is considerable variation in results, and particularly if there is inconsistency in the direction of effect, it may be misleading to quote an average value for the intervention effect.
3. *Explore heterogeneity.* It is clearly of interest to determine the causes of heterogeneity among results of studies. This process is problematic since there are often many characteristics that vary across studies from which one may choose. Heterogeneity may be explored by conducting subgroup analyses (see Section [10.11.3](https://training.cochrane.org/handbook/current/chapter-10#_Ref180060142)) or meta-regression (see Section [10.11.4](https://training.cochrane.org/handbook/current/chapter-10#_Ref180060159)). Reliable conclusions can only be drawn from analyses that are truly pre-specified before inspecting the studies’ results, and even these conclusions should be interpreted with caution. Explorations of heterogeneity that are devised after heterogeneity is identified can at best lead to the generation of hypotheses. They should be interpreted with even more caution and should generally not be listed among the conclusions of a review. Also, investigations of heterogeneity when there are very few studies are of questionable value.
4. *Ignore heterogeneity.* Fixed-effect meta-analyses ignore heterogeneity. The summary effect estimate from a fixed-effect meta-analysis is normally interpreted as being the best estimate of the intervention effect. However, the existence of heterogeneity suggests that there may not be a single intervention effect but a variety of intervention effects. Thus, the summary fixed-effect estimate may be an intervention effect that does not actually exist in any population, and therefore have a confidence interval that is meaningless as well as being too narrow (see Section [10.10.4](https://training.cochrane.org/handbook/current/chapter-10#_Ref180060192)).
5. *Perform a random-effects meta-analysis.* A random-effects meta-analysis may be used to incorporate heterogeneity among studies. This is not a substitute for a thorough investigation of heterogeneity. It is intended primarily for heterogeneity that cannot be explained. An extended discussion of this option appears in Section [10.10.4](https://training.cochrane.org/handbook/current/chapter-10#_Ref180060218).
6. *Reconsider the effect measure.* Heterogeneity may be an artificial consequence of an inappropriate choice of effect measure. For example, when studies collect continuous outcome data using different scales or different units, extreme heterogeneity may be apparent when using the mean difference but not when the more appropriate standardized mean difference is used. Furthermore, choice of effect measure for dichotomous outcomes (odds ratio, risk ratio, or risk difference) may affect the degree of heterogeneity among results. In particular, when comparator group risks vary, homogeneous odds ratios or risk ratios will necessarily lead to heterogeneous risk differences, and vice versa. However, it remains unclear whether homogeneity of intervention effect in a particular meta-analysis is a suitable criterion for choosing between these measures (see also Section [10.4.3](https://training.cochrane.org/handbook/current/chapter-10#_Ref180048248)).
7. *Exclude studies.* Heterogeneity may be due to the presence of one or two outlying studies with results that conflict with the rest of the studies. In general it is unwise to exclude studies from a meta-analysis on the basis of their results as this may introduce bias. However, if an obvious reason for the outlying result is apparent, the study might be removed with more confidence. Since usually at least one characteristic can be found for any study in any meta-analysis which makes it different from the others, this criterion is unreliable because it is all too easy to fulfil. It is advisable to perform analyses both with and without outlying studies as part of a sensitivity analysis (see Section [10.14](https://training.cochrane.org/handbook/current/chapter-10#_Ref180060260)). Whenever possible, potential sources of clinical diversity that might lead to such situations should be specified in the protocol.
